# Diagnostic Challenge of Necrotizing Soft Tissue Infection in a Patient With B Lymphocytic Leukemia

**DOI:** 10.7759/cureus.8387

**Published:** 2020-06-01

**Authors:** Andrius Sadauskas, Renata Jukneviciene, Valdas Pečeliūnas, Povilas Masionis, Giedrius Kvederas

**Affiliations:** 1 Trauma, Institute of Clinical Medicine, Clinic of Rheumatology, Orthopaedic Traumatology and Reconstructive Surgery, Centre of Orthopedics and Traumatology, Vilnius University Hospital Santaros Clinics, Vilnius University Faculty of Medicine, Vilnius, LTU; 2 Emergency Medicine, Institute of Clinical Medicine, Clinic of Emergency Medicine, Centre of Emergency Medicine, Vilnius University Hospital Santaros Clinics, Vilnius University Faculty of Medicine, Vilnius, LTU; 3 Internal Medicine, Institute of Clinical Medicine, Centre of Hematology, Oncology and Transfusiology, Vilnius University Hospital Santaros Clinics, Vilnius University Faculty of Medicine, Vilnius, LTU; 4 Orthopaedics, Institute of Clinical Medicine, Clinic of Rheumatology, Orthopaedic Traumatology and Reconstructive Surgery, Centre of Orthopedics and Traumatology, Vilnius University Hospital Santaros Clinics, Vilnius University Faculty of Medicine, Vilnius, LTU; 5 Orthopaedics, Clinic of Rheumatology, Orthopaedic Traumatology and Reconstructive Surgery, Centre of Orthopedics and Traumatology, Vilnius University Hospital Santaros Clinics, Vilnius University Faculty of Medicine, Vilnius, LTU

**Keywords:** necrotizing fasciitis, lrinec score, medical emergency, immunocompromised patient

## Abstract

The necrotizing soft tissue infection is a rare surgical emergency. Early diagnosis and timely treatment can contribute to better survival and the preservation of the limb. Diagnosis of this pathology remains a challenge to the clinician at the initial stage of the disease, especially if the patient is immunocompromised. We present the case of a 75-year-old man with B lymphocytic leukemia who was diagnosed with necrotizing soft tissue infection but failed to exhibit typical clinical and laboratory measurements. This case highlights the difficulty of early diagnosis in hematology patient, altered sensitivity of disease-specific scoring systems, and importance of high clinician awareness.

## Introduction

Necrotizing soft tissue infection (NSTI) is a rare life- and limb-threatening surgical emergency with rapid progression and devastating outcomes [1-4]. The disease could be described as a rapidly evolving infection of the fascia followed by secondary necrosis of the subcutaneous tissue and the skin with severe systemic inflammation and multiple organ damage [2-3]. The incidence of NSTI in the United States is estimated to be 500-1,500 cases per year or 0.04 cases per 1,000 person-years [5]. Different terms are used to classify the disease. Although, as the treatment and diagnostic approach is the same, different names only make diagnosis even more difficult, and we encourage the use of the term “necrotizing soft tissue infection” to encompass all of these infections [6].

This infection can damage all areas of the body, but usually the disease affects the limbs [7]. The involvement of extremities is often secondary to trauma, intravenous drug use, or insect bite, but NSTI can develop without any obvious portal of entry [6]. Certain conditions such as immunosuppression, diabetes, malignant tumors, and chronic thyroid disease are known risk factors [6-8].

The typical clinical sign of NSTI is the extreme and disproportional pain contrasting with slight dermal signs [2]. The Laboratory Risk Indicator for Necrotizing Fasciitis (LRINEC) score was introduced in 2004 and is based on laboratory parameters widely available across different institutions [9]. However, it has been recently criticized for its lack of ability to identify NSTI in early stages [10-12]. Though the diagnosis of NSTI at the initial stage of the disease remains a challenge, especially when a patient is immunocompromised, since the clinical expression is vague, the symptoms are non-specific and laboratory markers might be disguised.

## Case presentation

**Figure 1:**
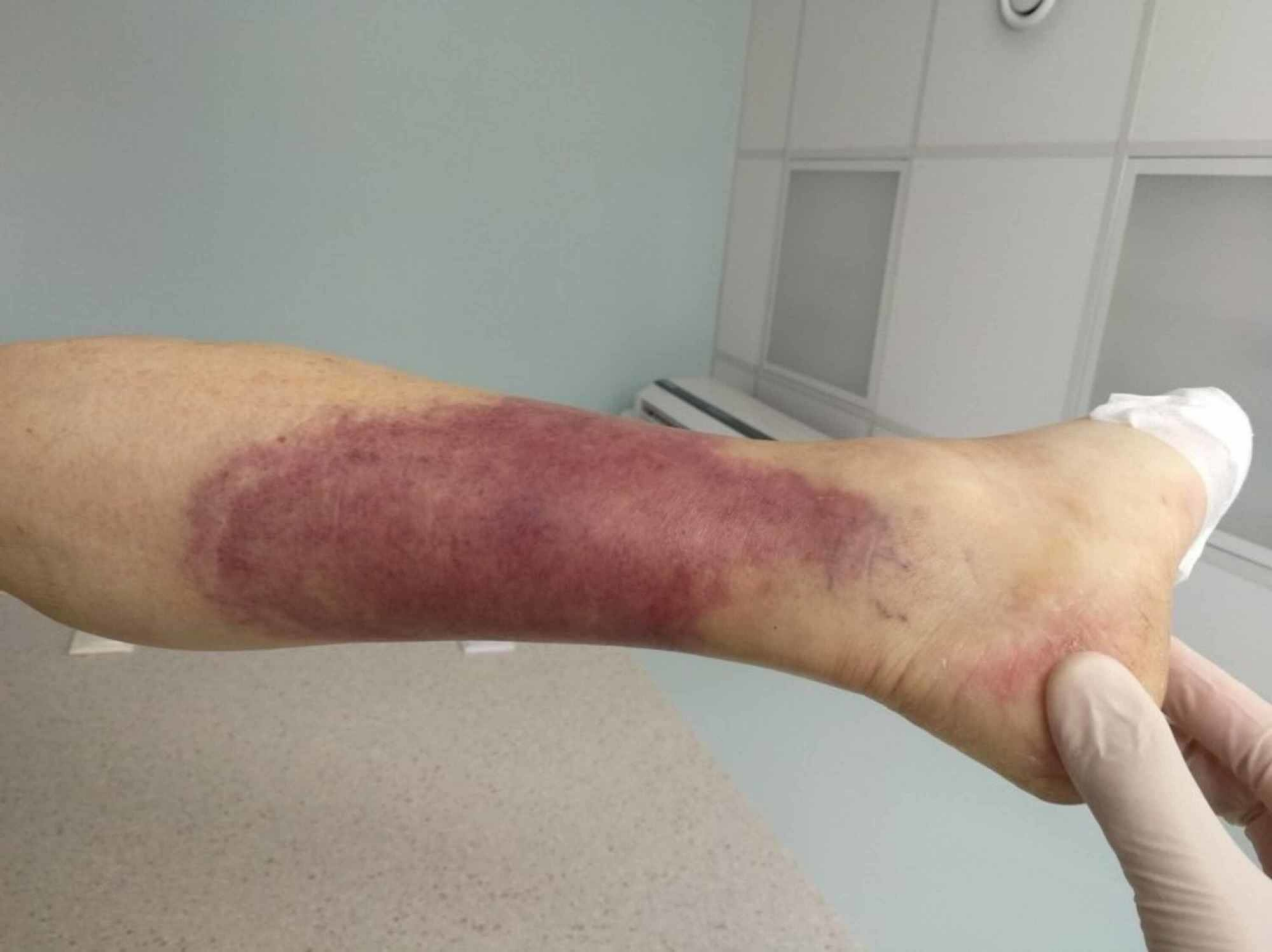
Erythematous region around the posterior surface of the right calf

**Figure 2:**
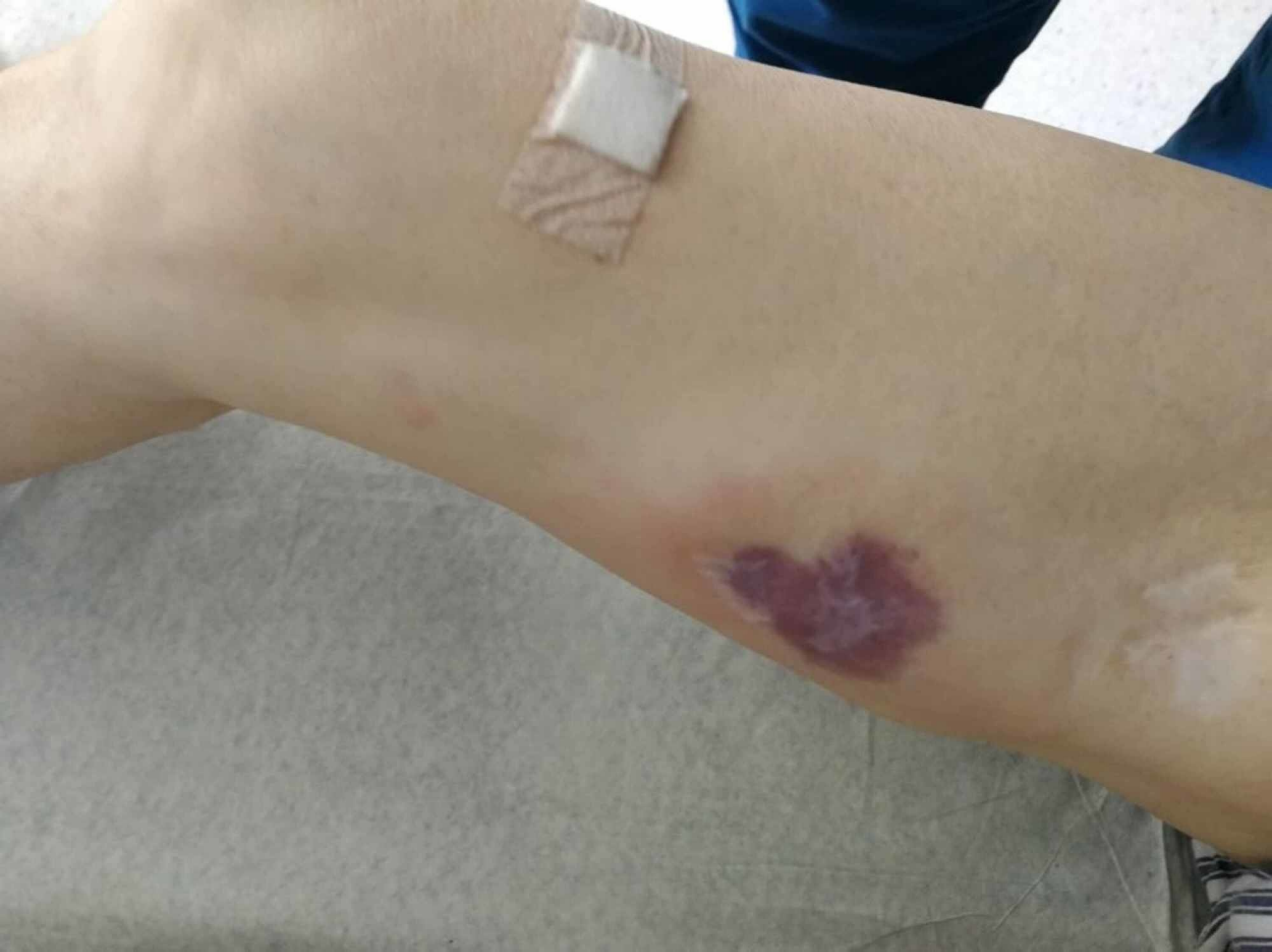
Erythematous region on the medial surface of the right thigh

A 75-year-old male stepped on a nail and presented to the Emergency Department where he got tetanus shot and wound care. The next day, he was febrile to 38.4°C, with swelling of the leg, and he presented for the second time. His white blood cell (WBC) count was normal and C-reactive protein (CRP) was 79 mg/L. Ultrasound of the soft tissue of the foot revealed subcutaneous tissue edema. The patient was prescribed oral antibiotics and sent home. However, third day after the trauma, he was brought by an ambulance due to severe pain, fever, and new bruising on his thigh and calf. On admission, the patient was pale and hypotensive. Inspection of the right leg revealed an erythematous swollen calf, with two small puncture wounds with discharge. There was an erythematous region without sharp margins around the posterior surface of the right calf (Figure 1), as well as on the medial surface of the thigh (Figure 2). Diffuse tenderness in his right leg was present. His medical history included B chronic lymphocytic leukemia (B-CLL), which was first diagnosed 14 years ago. Treatment for B-CLL was finished six months ago. The patient’s LRINEC score was 8, which strongly suggested the diagnosis of NSTI (Table 1).

**Table 1:** Comparison of the LRINEC and SIARI scores. LRINEC, Laboratory Risk Indicator for Necrotizing Fasciitis

LRINEC score			SIARI score	
Parameter	Range	Score	Parameter	Score
Hb (mg/L)	>135; 111-135; <111	0; 1; 2	Site of infection outside of the lower limb	3
White cells (10^9^/L)	<15; 15-25; >25	0; 1; 2	History of immunosuppression	3
Glucose (mmol/L)	≤ 10; >10	0; 1	Age ≤ 60 years	2
Sodium (mmol/L)	≥135; <135	0; 2	Creatinine (μmol/L) ≥ 141	1
Creatinine (μmol/L)	≤ 141; >141	0; 2	White cells (10^9^/L) ≥ 25	1
C-reactive protein (mg/L)	<150; ≥150	0; 4	C-reactive protein (mg/L) ≥ 150	1
Score ≤ 5 = <50% risk (low); 6–7 = 50% risk (intermediate); ≥8 = >75% risk (high).			Cutoff value is ≥3, with a sensitivity of 84% and a specificity of 70%.	

**Figure 3:**
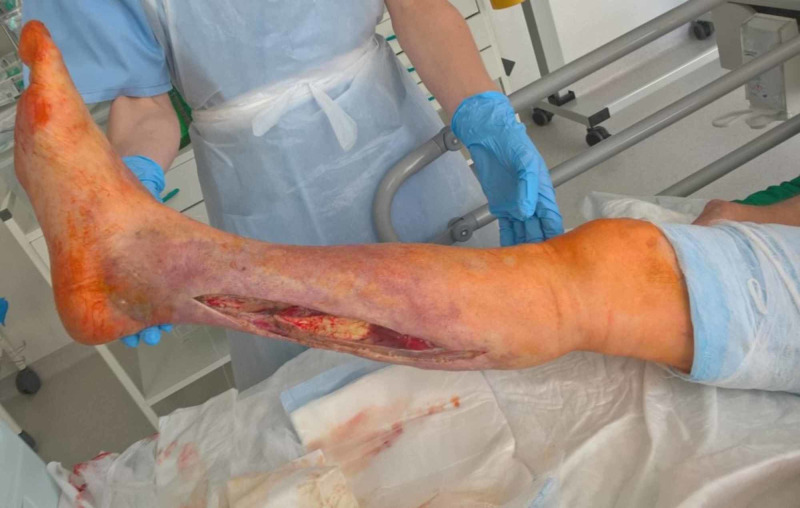
Necrotic greyish fascia in the posterior aspect of the wound

Emergency surgical debridement was performed within seven hours from the arrival. After incising the skin and exposing the fascia, a necrotic greyish fascia was seen (Figure 3). Histology sample revealed skin, subcutaneous tissue, and fascia necrosis. Complete surgical excision of all infected and necrotic tissue including a small margin of healthy fascia was performed. Empirical antibacterial treatment with meropenem, clindamycin, and vancomycin was initiated as recommended by the NSTI treatment guidelines [13,14]. Blood and wound culture showed growth of *Pseudomonas aeruginosa* sensitive to given antibiotics.

After surgery, the patient was treated in the intensive care unit due to sepsis and hemodynamic instability. Despite aggressive surgical debridement and targeted antibiotic therapy, cardiac and renal functions worsened, doses of vasopressors increased dramatically, and right foot cyanosis increased and reached up to the right knee during the first day. On the second day, because of continuously worsening condition, the decision was made to amputate the leg above the knee.

After the right leg amputation, his condition started to improve, he became hemodynamically stable, and he was transferred to the Traumatology Department within the next few days. Further treatment was uneventful, and the patient made full recovery.

## Discussion

Early diagnosis and timely treatment contribute to better survival rate as well as the preservation of the limb. Immunocompromised patients may fail to exhibit typical clinical and laboratory signs of NSTI [11]. Although our patient had B-CLL remission, on second admission he only had fever and tenderness of the foot, no classical symptoms of disproportional pain, and soft tissue ultrasound results were non-specific. Foo et al. described nine hematology patients with NSTI [15]. Although, eight of nine of these patients presented with the triad of typical symptoms, there was a delay of two to three days in diagnosis for three patients. To continue, most (75%) of these patients had an LRINEC score of <6 due to leukopenia, thrombocytopenia, and normal or mildly elevated CRP. In our case, the LRINEC score was 8 on the third admission, which, combined with emerged classical symptoms, strongly suggested the diagnosis of NSTI. Although we retrospectively analyzed the patient on the second visit, the LIRNEC score was 4, which stratifies a low risk of NSTI. In both admissions, WBC count was normal, but severe neutropenia was present too but neutrophils were not detected. This feature could be induced by B-CLL and might be a reason for false “normal”" WBC count and 1-2 points reduced LRNEC score on both admissions. This highlights that in hematology NSTI patients, the LRINEC score might fail to reach a cutoff value of ≥6. Cribb et al. recently proposed a novel decision support tool, the SIARI score, which was described as outperforming the LRINEC score [12]. Furthermore, it includes not only laboratory markers (CRP, creatinine, and WBC count) but also patient factors (site of infection, age, and immunosuppression history). In our case, the patient had a score of 3 at the second admission, which is a cutoff value for the SIARI score. We encourage the use of both LRINEC and SIARI scores as add-ons to diagnosis with caution as none of them can exclude NSTI, although clinical judgment and high index of suspicion remain the most important diagnostic tools (Table 1). To continue, we emphasize the importance of the fact that the patient came back to the Emergency Department for the second time; it is an unrecognized “symptom” of early NSTI and should raise physician awareness even more.

Whether we need to treat immunocompromised patients more aggressively remains the question of debate. A major proportion (55%) of these patients are hypotensive on presentation [15]. Furthermore, causative pathogen should be kept in mind as some of them are associated with higher mortality [16]. Decision to amputate should be made considering all the possible information about the patient and clinical findings. When treating those patients, the physicians should be very cautious and evaluate deteriorating clinical findings more often as laboratory work-up can be misleading.

## Conclusions

Early diagnosis combined with urgent antibacterial treatment, aggressive surgical debridement, and the joint work of emergency, surgical, and intensive care teams are cornerstones of a successful NSTI treatment. In immunocompromised patients, especially in hematology patients, delay of clinical presentation is common and disease-related changes in blood samples may deteriorate scoring systems. LRINEC and SIARI scores are important add-ons, but they should be used with caution in the decision-making process. In the end, early diagnosis of NSTI relies on physician awareness.
